# Ultrasound-guided Percutaneous Lavage in the Treatment of Calcific Tendinopathy of Elbow Extensor Tendons: A Case Report

**DOI:** 10.5704/MOJ.1607.011

**Published:** 2016-07

**Authors:** M Abate, V Salini, C Schiavone

**Affiliations:** University G d’Annunzio Chieti-Pescara, Chieti, Italy

**Keywords:** Calcific tendinopathy, elbow, ultrasound-guided, percutaneouslavage

## Abstract

We report the efficacy of the ultrasound-guided percutaneous treatment in the management of elbow extensor tendons calcific tendinopathy. The ultrasound-guided percutaneous treatment is broadly used with positive results in patients suffering from rotator cuff calcific tendinopathy. However, this interventional method has been reported only in one patient in the medical literature. A 34 years-old female who complained pain, swelling and severe functional limitation of the right elbow was referred to our unit. Elbow radiographs and ultrasound examination showed a soft-fluid calcification above the origin of the extensor tendons. Ultrasound-guided percutaneous treatment was therefore performed. After one year, the patient reported no pain and regained complete range of elbow motion. This method, in well trained hands, is an alternative treatment in the management of the uncommon elbow calcific deposit.

## Introduction

Elbow extensor tendon calcific tendinopathy is a rare condition, with an incidence of about 1% of elbow problems, characterized by the deposition of amorphous and globular calcium pyrophosphate crystals into extensors tendons. In the acute phase (calcific resorptive phase) it can be highly disabling with pain resistant to conventional therapies with analgesics, anti-inflammatory drugs, corticosteroids, physiotherapy, etc. When the conservative approach fails and function is impaired, surgery becomes mandatory.

The ultrasound-guided percutaneous treatment (UGPT) has already been performed with impressive results, in terms of pain reduction and functional improvement, in the short, mid and long terms, in patients suffering from rotator cuff calcific tendinopathy^1-3^. This interventional procedure is an elective technique, minimally invasive and not painful, of low-cost, and requires, in well-trained hands, short procedure time. However, to our knowledge, this method has been attempted only in one case in elbow extensors tendon calcific tendinopathy after the failure of conservative treatments^4^.

The aim of this case report is to describe the results of the UGPT as an alternative treatment in the management of a single case of uncommon elbow extensor tendons calcific tendinopathy.

## Case Report

**Fig. 1:**
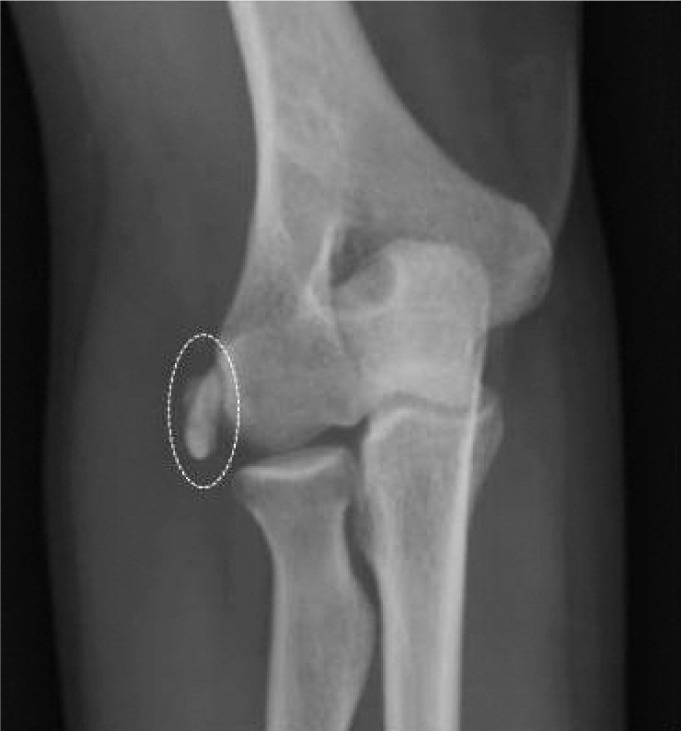
Anterior-posterior radiograph of right elbow, showing the presence of a large calcification in the soft tissues near the lateral epicondyle.

**Fig. 2:**
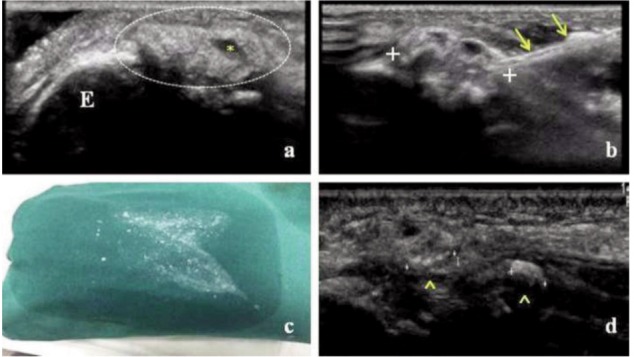
At baseline, the US exam showed an homogeneous and hyperechoic calcific deposit (circle) with few anechoic areas inside (*) **(A)** During the UGPT two needles (arrows) were inserted into the calcification (calipers) which was then washed **(B)** At the end of UGPT, which lasted about ten minutes, small calcific fragments were visible on the sterile cloth **(C)** At 4 weeks the US control showed only small hard calcific foci in the subcutaneous tissue (calipers, arrowheads) **(D)** E= lateral epicondyle.

A 34 years-old female working in a light occupation, complaining of pain, swelling and severe functional limitation (range of motion [ROM] 75-120°) of the right elbow attended our clinic. She reported that the symptoms developed several weeks previously without any specific injury. The physical examination revealed a hard swelling on the lateral side of the joint, and a radiograph revealed a calcific deposit near the lateral epicondyle (Figure 1). Little relief was achieved with conservative therapies with rest, ice pack and NSAIDs prescribed by other physicians. After one month, due to the patient’s worsening symptoms, she was referred to our Echographic Unit. We performed an ultrasound examination which revealed the presence of a soft-fluid calcific image (3 x 1cm) in the subcutaneous tissue above the extensors tendons origin which however, was not involved. The deposit appeared homogeneous and hyperechoic with few anechoic areas inside without posterior acoustic shadow (resorptive phase) (Figure 2a).

Based on the clinical symptoms and the imaging examinations the UGPT was proposed and performed^1^ after obtaining informed written consent from the patient. Briefly, under sterile conditions, after subcutaneous and pericalcific anesthesia (10 ml of 2% mepivacaine chloridrate), two 18G needles were inserted into the calcific deposit. Once verified their correct placement, one of the needles was connected to a 20 mL luer lock syringe through a 3-ways valve; calcification was then irrigated with NaCl 0.9% saline solution which allowed the breaking, dissolution and elimination of calcium through the other needle (Figure 2b). The lavage was repeated several times (about 5-6) until the flushed fluid was completely free of visible calcium. At the end of the procedure, which lasted about ten minutes, small calcific fragments were visible on the sterile cloth (Figure 2c). On discharge, we suggested applying ice packs at home; antibiotics for five days were prescribed and analgesics to be taken as required. The patient was advised to follow a rehabilitation protocol (passive and active ROM exercises).

At review at one month, the patient reported only slight pain, especially on making a strong grip, and had complete full range of motion in the elbow; no adverse events had been observed after treatment and he had not taken the analgesic. Ultrasound examination showed only small hard calcific focus (few millimeters) in the subcutaneous tissue (Figure 2d), which however did not require any further treatment. At review at one year the patient no longer complained of elbow pain and had resumed normal activity of daily living. The ultrasound image was unchanged and elbow radiographs were not carried out.

## Discussion

This case report shows that the UGPT, which is widely performed with positive results in the treatment of rotator cuff calcific tendinopathy, can be used also in the management of the uncommon elbow calcific deposit. Our findings are in agreement with De Zordo *et al* who reported positive results in one patient suffering from elbow calcification after the ultrasound-guided lavage using a two-needle technique with 16-18 G needles^4^. Apart the impressive clinical outcomes, the radiographic and ultrasound exams, aiming to evaluate the size and quality of the calcification and alterations of the tendon and surrounding tissues, performed before and six weeks after the procedure, showed a significant calcium reduction without any adverse events. We also observed after one month and one year follow-up only few small and hard calcific specks in the subcutaneous tissue on the ultrasound evaluation.

The mechanism of action of the UGPT is very simple: it allows, under local anesthesia, by means of the saline solution the breaking, dissolution and elimination of the calcific deposits. Patients suffering from calcific tendinopathy in the resorptive phases (which is the most painful) can mainly benefit from this treatment while the results are less impressive when treating hard calcifications (minor limitation of the procedure)^4^. Few complications (subacromial-subdeltoid adhesive bursitis, tenosynovitis, tendon ruptures) have been observed in the management of rotator cuff calcific tendinopathy^1-4^. We, however, did not observe any adverse events in this case report.

In conclusion, our result is of clinical relevance because it shows that the UGPT can be an alternative therapy for elbow extensor tendon calcific tendinopathy, avoiding the known side effects (gastrointestinal, renal, hepatic and cardiovascular) of the common pharmacological treatments. This procedure is of low-cost, non-invasive and minimally painful, and requires in well trained hands (physicians with a strong background in ultrasound-guided procedures), short procedure time.
